# Electrodiffusive Model for Astrocytic and Neuronal Ion Concentration Dynamics

**DOI:** 10.1371/journal.pcbi.1003386

**Published:** 2013-12-19

**Authors:** Geir Halnes, Ivar Østby, Klas H. Pettersen, Stig W. Omholt, Gaute T. Einevoll

**Affiliations:** 1 Department of Mathematical Sciences and Technology, Norwegian University of Life Sciences, Ås, Norway; 2 Centre for Integrative Genetics, Department of Mathematical Sciences and Technology, Norwegian University of Life Sciences, Ås, Norway; 3 Centre for Integrative Genetics, Department of Animal and Aqucultural Sciences, Norwegian University of Life Sciences, Ås, Norway; Indiana University, United States of America

## Abstract

The cable equation is a proper framework for modeling electrical neural signalling that takes place at a timescale at which the ionic concentrations vary little. However, in neural tissue there are also key dynamic processes that occur at longer timescales. For example, endured periods of intense neural signaling may cause the local extracellular K^+^-concentration to increase by several millimolars. The clearance of this excess K^+^ depends partly on diffusion in the extracellular space, partly on local uptake by astrocytes, and partly on intracellular transport (spatial buffering) within astrocytes. These processes, that take place at the time scale of seconds, demand a mathematical description able to account for the spatiotemporal variations in ion concentrations as well as the subsequent effects of these variations on the membrane potential. Here, we present a general electrodiffusive formalism for modeling of ion concentration dynamics in a one-dimensional geometry, including both the intra- and extracellular domains. Based on the Nernst-Planck equations, this formalism ensures that the membrane potential and ion concentrations are in consistency, it ensures global particle/charge conservation and it accounts for diffusion and concentration dependent variations in resistivity. We apply the formalism to a model of astrocytes exchanging ions with the extracellular space. The simulations show that K^+^-removal from high-concentration regions is driven by a local depolarization of the astrocyte membrane, which concertedly (i) increases the local astrocytic uptake of K^+^, (ii) suppresses extracellular transport of K^+^, (iii) increases axial transport of K^+^ within astrocytes, and (iv) facilitates astrocytic relase of K^+^ in regions where the extracellular concentration is low. Together, these mechanisms seem to provide a robust regulatory scheme for shielding the extracellular space from excess K^+^.

## Author Summary

When neurons generate electrical signals they release potassium ions (K^+^) into the extracellular space. During periods of intense neural activity, the local extracellular K^+^ may increase drastically. If it becomes too high, it can lead to neural dysfunction. Astrocytes (a kind of glial cells) are involved in preventing this from happening. Astrocytes can take up excess K^+^, transport it intracellularly, and release it in regions where the concentration is lower. This process is called spatial buffering, and a full mechanistic understanding of it is currently lacking. The aim of this work is twofold: First, we develop a formalism for modeling ion concentration dynamics in the intra- and extracellular space. The formalism is general, and could be used to simulate many cellular processes. It accounts for ion transports due to diffusion (along concentration gradients) as well as electrical migration (along voltage gradients). It extends previous, related formalisms, which have focused only on intracellular dynamics. Secondly, we apply the formalism to model how astrocytes exchange ions with the extracellular space. We conclude that the membrane mechanisms possessed by astrocytes seem optimal for shielding the extracellular space from excess K^+^, and provide a full mechanistic description of the spatial (K^+^) buffering process.

## Introduction

The interaction between neurons and glial cells has been the topic of many recent studies within the field of neuroscience (see reviews in [1]–[3]). Astrocytes (a species of glial cells) play an important role in modulating excitatory and inhibitory synapses by removal, metabolism, and release of neurotransmitters [4], homeostatic maintenance of extracellular K^+^, H^+^, and glutamate [5], supply of energy substrates for neurons [6], and neuronal pathfinding during development and regeneration [7]. Astrocytic cells seem to have key roles in many central nervous system disorders, ranging from neuropathic pain and epilepsy to neurodegenerative diseases such as Alzheimers, schizophrenia and depression [8]. Computational models of neuron-glia interactions is a prerequisite for understanding the dysfunctional situations, and for assessing glial cells as a potential therapeutic target [9]. To give a few examples, such models have been used to simulate glial regulation of extracellular K^+^-concentration [10]–[13], and the relation between extracellular K^+^-dynamics and epileptic seizures [14]–[16] and spreading depression [17], [18].

Regulation of the extracellular K^+^-concentration is considered one of the key cellular functions of astrocytes [2]. During normal conditions, the extracellular K^+^-concentration (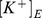) is typically maintained close to the baseline level (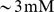). However, when neurons fire action potentials, they expel K^+^ into the extracellular space. During periods of intense neural activity, the local extracellular K^+^-concentration may increase by several millimolars, and may interfere with neural activity [10], [19], [20]. Concentrations between 8 and 12 mM are often considered a limit to pathological conditions [3], [12], [21].

Orkand (1966) [22] discovered that astrocytes can funnel out excess K^+^ from high concentration regions by a process coined spatial buffering [12], [21], [22]. According to this concept, K^+^ is taken up by the glial cell from high-concentration sites, evoking a local depolarization of the glial membrane. K^+^ is then transported longitudinally inside the glial cell (and possibly through several glial cells connected by gap junctions into a glial syncytium [10], [23]), and eventually expelled into the ECS at more distal cites where 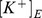 is lower. However, it has also been argued that astrocytes may reduce 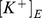 by local uptake and temporal storage, not necessarily including transport over distances [19], [24]. Furthermore, diffusion through the ECS is also involved in transporting excess K^+^ out from high concentration regions. The relative importance of these different clearance mechanisms are under debate [25].

Electrical neural signalling is typically modeled using the cable equation, where dendrites and axons are represented as one-dimensional, possibly branching, electrical cables, and the transmembrane potential is the key dynamical variable [26], [27]. With the possible exception of the signalling molecule Ca^2+^ (see e.g., [28], [29]), ion concentrations are typically assumed to be constant. The effect of ionic diffusion (due to concentration gradients) on the net electrical currents is neglected in standard cable theory, and resistivities (which in reality depend on ion concentrations) are assumed to be constant. These are often good approximations, as concentrations of the main charge carriers (K^+^, Na^+^ and Cl^−^) in the extracellular- (ECS) or intracellular space (ICS) typically vary little at the short time-scale relevant for electrical neural activity (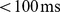).

Glial function typically involves processes that take place at a longer time-scale (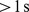), at which significant variations in ionic concentrations may occur. For example, the process of spatial K^+^-buffering involves local uptake, a local depolarization of the astrocytic membrane, and longitudinal electrodiffusive transports through the intracellular- (ICS) and extracellular space (ECS) propelled both by voltage- and concentration gradients [30]. A mechanistic understanding of glial function thus requires a modelling scheme that in a consistent way can capture the intricate interplay between ion concentration dynamics and the dynamics of 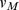. Physically, 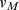 is determined by the total electrical charge on the inside (or outside) of the membrane, which in turn is uniquely determined by the concentrations (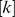) of all ionic species that are present there [31]. In some heart cell models, ion concentrations have been reported to drift to unrealistic values in long-term simulations, while 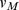 maintain realistic values [32]–[34]. Whether the relationship between 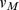 and 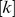 is consistent, is a general concern with models that explicitly depend on both. If applied to general problems, and in particular in long-term simulations, models that do not ensure an internally consistent 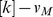 relationship may give erroneous predictions.

**Figure 1:**
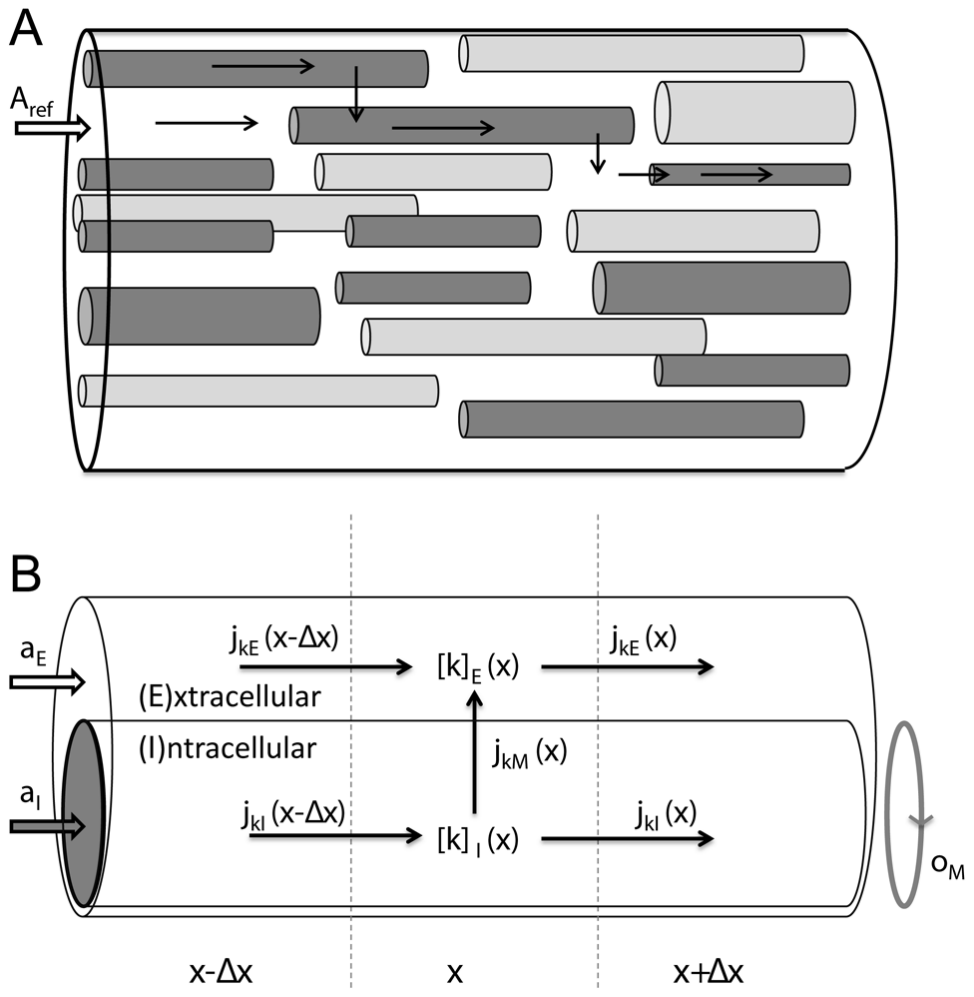
A two domain-model for ion concentration dynamics in the intra- and extracellular space, when macroscopic transport is essentially one-dimensional. (A) A piece of neural tissue with cross section area 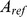 and an arbitrary extension 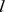 in the 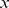-direction. The tissue contains cells (dark grey) that participate in the transport process, and cells that do not (light grey). (B) The interior of all participatory cells represented as a single, equivalent cylindrical cable (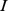), coated by ECS (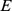). The geometry is specified by three parameters, where 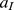 and 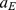 are, respectively, the fractions of 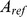 occupied by the ICS of participatory cells and the ECS, and 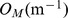 is the amount of membrane area per tissue volume (or, equivalently, the circumference of the equivalent cable divided by 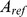). Due to the presence of other cells (non-participatory), we generally have that 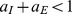. The concentration of ion species 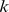 is denoted 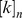 where 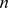 represents domain 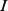 or 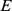. Ionic movement is described by the transmembrane flux density (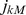) and the longitudinal flux densities due to electrical migration (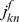) and diffusion (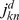).

Gardner-Medwin (1983) [10] proposed a pioneering computational model of the spatial buffering process, later re-analyzed by Chen and Nicholson (2000) [12]. In this model, spatial buffering was considered as an essentially one-dimensional transport process. The complex composition of the tissue (Fig. 1*A*) could then be simplified to a two-domain model as that illustrated in Fig. 1*B* [10], [12]. There, the ICS of all cells participating in the transport process (i.e. the astrocytes) have been represented as an equivalent cable (*I*-domain) which is coated by ECS (*E*-domain). The *I*-*E* system could be pictured phenomenologically as an representative single astrocyte, coated with the average proportion of available ECS per astrocyte. This geometrical simplification was motivated for one-dimensional transport phenomena through the glial syncytium [10], [12], but could in principle apply to any transport phenomena that justifies a geometrical simplification as that in Fig. 1. A limitation with these modelling studies [10], [12], and related modelling studies by Newman and coworkers [11], [21], is that 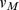 was derived from standard cable theory, which neglects effect from diffusive currents on 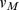. The concern regarding a consistent relationship between 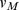 and the ionic concentrations thus also applies to these models.

Qian and Sejnowski (1989) have previously developed a consistent, electrodiffusive scheme for modelling the dynamics on 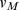 and ion concentrations [31]. Like the standard cable model, the electrodiffusive model assumes that transport phenomena are essentially one-dimensional. Unlike the standard cable model, the electrodiffusive model derived 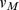 from the ion concentration dynamics, accounting for all ionic movements (membrane fluxes, longitudinal diffusion, and longitudinal electrical migration), as well as for the concentration-dependent variation of the intracellular resistivities. An important limitation with this previous electrodiffusive model is that it only includes intracellular dynamics, whereas the ECS was assumed to be isopotential and with constant ion concentrations [31]. This was a useful simplification for simulating a small intracellular compartment, such as a dendritic spine [31], but is not generally applicable to macroscopic transport mechanisms. In particular, it can not be applied for modelling the spatial buffering process, where ion concentration dynamics in the ECS plays a paramount role. In reality, the ECS comprises about 20% of the total neural tissue volume, while the remaining 80% is the ICS of various cells [12]. When a large number of cells participate in simultaneous ion exchange with the ECS, the impact on the ion concentrations in the ICS and ECS may be of the same order of magnitude.

The aim of this work is twofold: First, we generalize the electrodiffusive formalim [31] to a explicitly include the ECS. The result is a general mathematical framework for consistently modelling the dynamics of the membrane potential (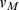), the intra- (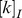) and extracellular (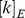) ion concentrations for a set (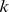) of ionic species. We believe that this framework will be of general value for the field of neuroscience, as it can be applied to any system that justifies a geometrical description as that in Fig. 1*B*. Next, we apply the electrodiffusive formalism in a spatially explicit model of astrocytes exchanging ions with the ECS. We run simulations to investigate the efficiency of the spatial K^+^-buffering process, relative to that of local uptake/storage by astrocytes, and that of diffusion in the ECS alone. Unlike the previous models [10]–[12], [21], our astrocyte model is based on the prevailing view that Na^+^/K^+^/ATPase-pump is the main uptake mechanism for K^+^ [3]. Furthermore, as our model was based on a physically consistent electrodiffusive formalism, we arrive at a full mechanistic description of the buffering process, which quantitatively describes the intricate interplay between 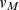 and the dynamics of ion concentrations.

This article is organized in the following way: The Model section contains two main parts. In the first part, we present the electrodiffusive formalism for computing the ion concentration dynamics in a system described by the geometry depicted in Fig. 1*B*. We consider this theoretical framework a key contribution of this work. However, the key concepts introduced in this part are summarized in Table 1, and with this in hand, the reader who is mainly interested the biological process of spatial K^+^-buffering by astrocytes may therefore skip to second part of the Model-section. There, the model for astrocytes exchanging ions with the ECS is presented. The Results section is devoted to simulations on the astrocyte model, and provides an improved biophysical insight in the electrodiffusive mechanisms utilized by astrocytes to spatially buffer K^+^. By comparing different versions of the model, we also assessed the importance of spatial buffering, relative to that of other clearance mechanisms such as local uptake/storage by astrocytes and diffusion through the ECS alone. Finally, in the Discussion section we address how our mathematical framework relates to previous electrodiffusive modeling frameworks. We also summarize the new insights that our simulations have given in the process of spatial K^+^-buffering by astrocytes.

**Table 1:** List of symbols and definitions.

Symbol	Explanation	Units
*k* (index)	Ion species: 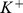 , 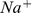 or 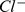	
*n* (index)	Domain: *I* (ICS) or *E* (ECS)	
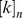	Ion concentration of species *k* in domain *n*	mM
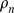	Charge density	C/m^3^
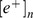	Charge density, represented as concentration of unit charge	mM
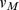	Membrane potential	mV
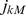	Membrane flux density of species *k*	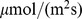
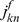	Axial flux density due to electrical migration	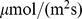
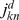	Axial flux density due to diffusion	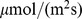
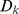	Diffusion constant in diluted media	m^2^/s
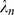	Tortuosity (effective diffusion constant = 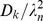 )	
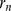	Resistivity	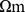
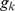	Membrane conductance for passive ion channels	S/m^2^
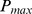	Maximum Na^+^/K^+^ pump-rate	mol/(m^2^s)
*l*	Length of astrocyte	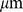
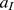	Astrocyte volume/total tissue volume	
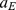	ECS volume/total tissue volume	
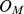	Membrane area/total tissue volume	m^−1^
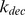	Rate for concentration dependent output	m/s
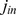	Constant input flux density in input zone	mol/(m^2^s)

## Model

### Electrodiffusive formalism

In Fig. 1*B*, particles in *I* or *E* may move along the *x*-axis or across the membrane. In a segment 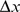 of *I*, centered at *x*, and with volume 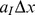, the particle concentration dynamics of an ion species 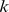 is determined by:
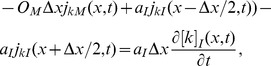
(1)where the transmembrane- (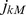), the intracellular- (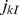) and the extracellular (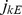) flux densities of particle species 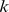, have units mol/(m^2^s). The first term on the left represents the ionic flux that enter this segment through the piece of the membrane with area 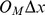. The negative sign follows from 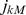 (by convention) being defined as positive in the direction from *I* to *E*. The second and third terms represent the ionic fluxes that enter(+)/leave(−) the section through the left/right boundaries, with cross section areas 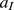. If the net flux into the segment is nonzero, the ion concentration will build up over time, according to the right hand side of Eq. 1.

We divide Eq. 1 by 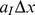, and take the limit 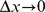, to obtain the continuity equation on differential form:
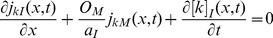
(2)

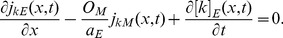
(3)We have also written up the continuity equation for the extracellular domain.

The axial flux densities are described by the generalized Nernst-Planck equation:
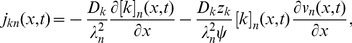
(4)where 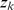 is the valence of ion species 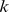, and the index *n* represents *I* or *E*. The first term on the right in Eq. 4 is the diffusive flux density (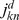), driven by the concentration gradients, and the last term is the field flux density (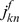), i.e., the flux density due to ionic migration in the electrical field. The effective diffusion constant 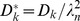 is composed of the diffusion constant 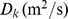 in dilute solutions and the tortuosity factor 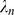, which summarizes the hindrance imposed by the cellular structures [12], [35]. We use 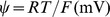, where 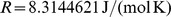 is the gas constant, 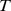 the absolute temperature, and 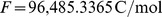 is Faraday's constant.

The formalism is general to the form of 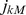, which may include contribution from multiple membrane mechanisms, such as ion pumps, co-transporters and ion channels. It is sufficient to require that 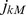 is known at any point in time given the voltage across the membrane, the ionic concentrations on either side of the membrane, and possibly some additional local information (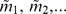) reflecting the local state of the membrane:
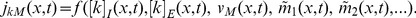
(5)


As boundary conditions, we shall apply the sealed-end condition, i.e., we assume that no fluxes enter or leave through the ends (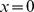 and 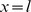) of *I* or *E*:
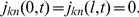
(6)


Equations 2–3, together with with Eqs. 4, 5 and 6, specify the system we want to solve. Before we derive the electrodiffusive formalism for this problem, we recall how the standard cable equation can be derived from the principles of particle conservation.

#### Charge conservation

The particle conservation laws (Eqs. 2–3) can be transformed to charge conservation laws by the use of the general relations (see e.g., [27]):
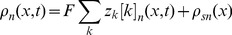
(7)

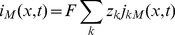
(8)

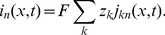
(9)Here, 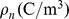 is the charge density, 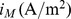 is the transmembrane current density, and 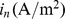 is the axial current density. For practical purposes, we have included a density of static charges (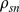) in Eq. 7, representing contributions from ions/charged molecules that are not considered in the conservation equations. If the set 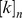 include all present species of ions, then 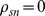. To keep notation compact, we from here on omit the functional arguments 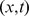.

If we multiply the particle conservation laws (Eqs. 2–3) by 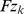, take the sum over all ion species, *k*, and use Eqs. 7–9, we obtain the equivalent laws for charge conservation:
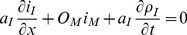
(10)

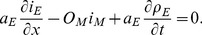
(11)Note that the last term only depends on the mobile ions, as 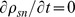.

#### Standard cable equation

The standard cable equation may be derived by combining the charge conservation laws (Eqs. 10–11) with three simplifying assumptions: (i) *E* is assumed to be isopotential and with zero resistivity, (ii) the membrane is a parallel-plate capacitor, and (iii) ion concentrations are effectively constant, i.e., diffusive currents are negligible and resistivities (see Eq. 15 below) are constant.

Assumption (i) implies that we only need to consider charge conservation in *I* explicitly. To obtain the cable equation in the standard form, we must express 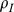 and 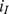 in Eq. 10 in terms of 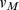 and 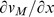.

Assumption (ii) allows us substitute 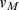 for 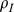. A capacitor with capacitance 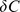 separates a charge 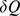 from the opposite charge 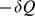, and generates a voltage difference 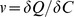. The charge inside a piece (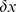) of membrane with area 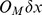 is 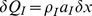. The capacitance of this piece of membrane is 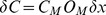, where 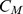 denotes the membrane capacitance per membrane area. We therefore obtain:
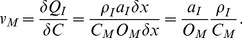
(12)


According to assumption (iii), diffusive currents are negligible, and Eq. 4 reduces to:
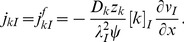
(13)If we insert Eq. 13 into Eq. 9, we see that the axial current density obeys Ohm's current law:
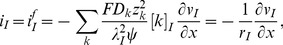
(14)where we have identified the resistivity, 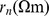:
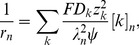
(15)in the ICS (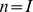). Finally, we insert Eqs. 14 and 12 into Eq. 10 to obtain the cable equation:
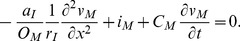
(16)Note that 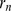 generally depends on 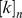. However, we have here assumed that 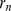 is constant (cf. assumption (iii)). Furthermore, we have used the identity: 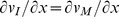, which follows from the definition
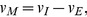
(17)together with the assumption (i) that *E* is isopotential. Eq. 16 is the most commonly used form of the cable equation, although there are versions that also explicitly considers spatiotemporal variations of the potential in the extracellular domain [26].

#### Two-domain electrodiffusive model

The cable equation only considers the net electrical transports, and “hides” the underlying transports of different ionic species. We now develop the electrodiffusive formalism for computing the ion-concentration dynamics. Like in standard cable theory, we limit the study to the one-dimensional geometry in Fig. 1*B*. Unlike standard cable theory, we explicitly consider both domains *I* and *E*, and we do not neglect diffusive currents nor concentration dependent variations of the resistivities.

The conservation equations (Eqs. 2–3), with the Nernst-Planck equation (Eq. 4) for 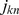 specify the system we want to solve. As in standard cable theory, the formalism is general to the form of 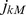 (Eq. 5). With 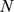 ion species, Eqs. 2–3 represent a system of 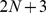 variables which are functions of 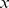 and 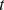. These are the 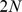 concentration variables (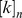 for 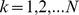 and 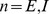), and the three additional variables (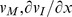 and 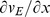) occurring in the expressions for the flux densities.

To reduce the number of independent variables to the 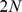 state variables (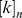) we need three conditions relating 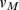, 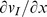 and 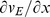 to 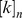. The first two conditions we recognize from standard cable theory, while the third is new:

- C1: 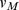 is determined by the charge density (Eq. 12).
- C2: 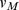 is defined as 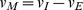 (Eq. 17).
- C3: The charge densities in 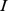 and 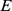 fulfill the *charge symmetry condition* (Eg. 18).

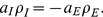
(18)We here explain the origin of C3. According to condition C1, 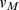 is given by:
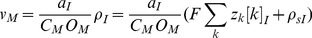
(19)where we have inserted Eq. 7 for 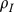, so that 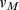 is expressed in terms of ionic concentrations. Equivalently, we may also express 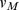 in terms of the ion concentrations in the ECS:
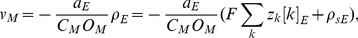
(20)where the negative sign follows from the convention that 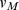 is positive when 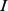 is positively charged. By demanding consistency between Eq. 19 and Eq. 20, we can derive the *charge symmetry condition* (Eq. 18), which states that the charge on the inside of a piece of membrane is equal in magnitude and opposite in sign to the charge on the outside. C1 and C3 are both implicit when the membrane is assumed to be a parallel plate capacitor. C3 is also related to the issue of electroneutrality (see Discussion).

The next step is to express the voltage gradients (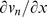) in terms of ionic concentrations. The constraints C2 (Eq. 17) and C3 (Eq. 18) allow us to derive two independent equations that relate 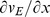 and 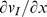. The first equation is obtained by differentiating Eq. 17:
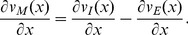
(21)We recall that 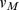 is already a known function of ion concentrations (Eq. 19 or Eq. 20).

A second equation relating 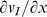 to 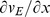 may be derived by combining Eq. 18 with the charge conservation laws. If we sum Eqs. 10 and 11, we immediately see that the terms involving 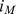 cancel out. Due to Eq. 18, also the last terms on the left cancel, so that we are left with:
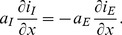
(22)Due to sealed end-condition (Eq. 6), 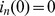, so that Eq. 22 takes the simple form:
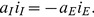
(23)If the charge symmetry condition (C3) is satisfied at a given time 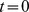 (and we must specify the initial concentrations so that this is true), Eq. 23 is the condition that it remains satisfied at all times 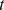.

We now decompose the current density into a diffusive term and a field term: 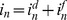, and express 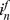 in terms of Ohm's law (cf. Eq. 14). If we insert this into Eq. 23, we obtain the second equation relating 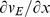 and 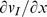:
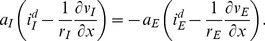
(24)


**Figure 2:**
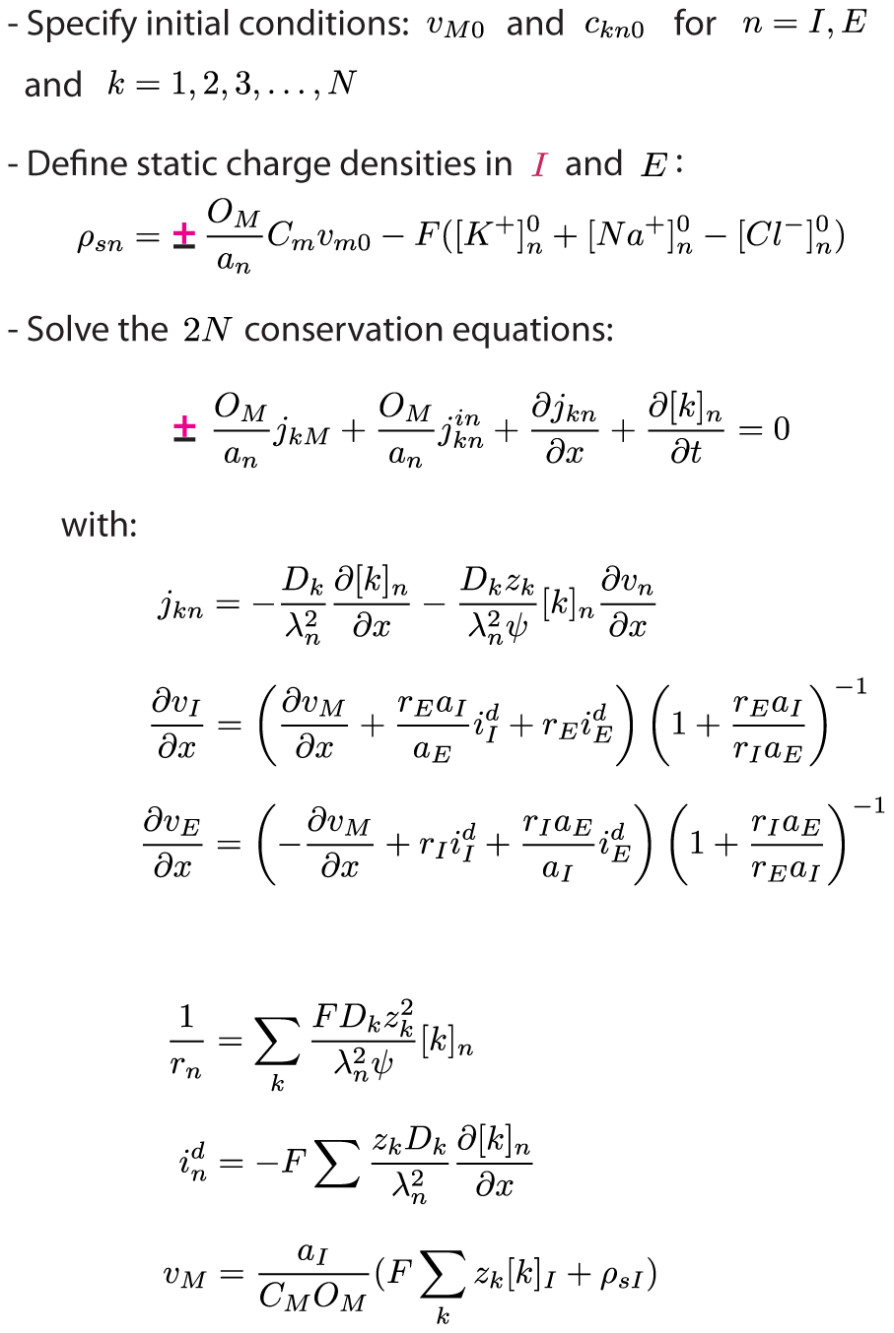
Summary of the two-domain electrodiffusive formalism. The set of equations summarizes the electrodiffusive formalism. In equations containing the symbol “±”,“+” should be used for intracellular domain (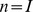) and “−” should be used for the extracellular domain (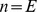). The formalism is general to the choice of membrane mechanisms. 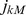, representing system specific membrane mechanisms (ion pumps, ion channels, cotransporters ect.), must to be specified by the user. External input to the system must also be specified. The input must be locally electroneutral, i.e., must fulfill 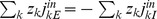.

Finally, Eq. 21 and Eq. 24 can be solved for the voltage gradients. After some simple algebra we obtain:
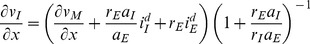
(25)

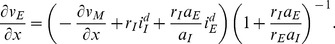
(26)Here, 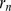 is given by Eq. 15, 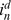 by Eq. 4, and 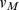 by Eq. 19 or Eq. 20. All voltage terms are thereby expressed in terms of ionic concentrations. With this, the conservation equations (Eqs. 2–3) are fully specified, and can be solved numerically with appropriate boundary conditions. The final set of equations is summarized in Fig. 2.

#### External input to the electrodiffusive model

As we have indicated in Fig. 2, an external input to the system can be incorporated in the formalism by adding terms 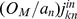 to the left hand sides of Eqs. 2 and/or 3. In order not to invalidate the charge symmetry condition (C3), such an input needs to fulfill the relation:
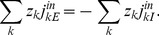
(27)This means that input current density into the ECS and ICS must have the same absolute value and the opposite sign (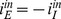), so that no net electrical current enters the system at a given 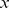. If only one domain receives external input, Eq. 27 reduces to:
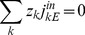
(28)To give a practical, illustrative example, let us assume that we want to inject a K^+^-influx to the ECS (as we later do in the astrocyte/ECS-model). We would then add the term 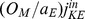 to the left hand side of Eq. 3 (the version where 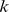 represents K^+^). To fulfill Eq. 28, such an external influx of cations would need to be compensated by a corresponding efflux of cations of another species (e.g, Na^+^), or a corresponding influx of anions (e.g., Cl^−^), or a combination of the two. In the astrocyte model we applied the former, i.e., we defined 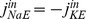. This was implemented by adding the term 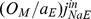 to the left hand side of Egn. 3 (the version where 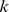 represents Na^+^).

#### Electrodiffusive formalism vs. cable equation

From Eq. 10, following from charge conservation in *I*, we may derive a differential equation for the dynamics of 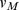. We use Eq. 19 to substitute 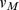 for 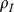. Furthermore, we use the decomposition 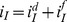, with Eq. 14 for 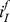, and Eq. 25 for 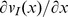. We then obtain:
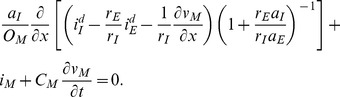
(29)This is the equivalent to the standard cable equation (Eq. 16), for the electrodiffusive two-domain system.

A few notes: Firstly, a corresponding dynamical equation for 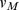 could have been derived from the extracellular conservation law (Eq. 11). Due to the charge symmetry condition, the two equations would be equivalent. Secondly, unlike the standard cable equation, Eq. 29 does not provide a complete system description, as Eqs. 2–3 must be solved to determine 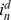 and 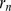. Thirdly, when the ionic concentrations are known, Eq. 29 is not necessary for computing 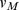, as 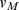 can be computed algebraically from Eq. 19. Eq. 29 is mainly useful for comparison with the standard cable equation.

We can immediately see that if we make the common assumptions (i) that the extracellular resistivity (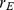) is zero, (ii) that the diffusive currents (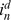) are zero, and (iii) that the intracellular resistivity (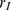) is constant, then Eq. 29 reduces to the standard cable equation (Eq. 16). We should note that there are two-domain versions of the cable equation where the first assumption is not made [26]. The two other assumptions are warranted only in cases when the spatiotemporal variations in ionic concentrations is such that 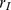 varies little, and 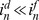 during the time course of a simulation.

### Astrocyte model

**Figure 3:**
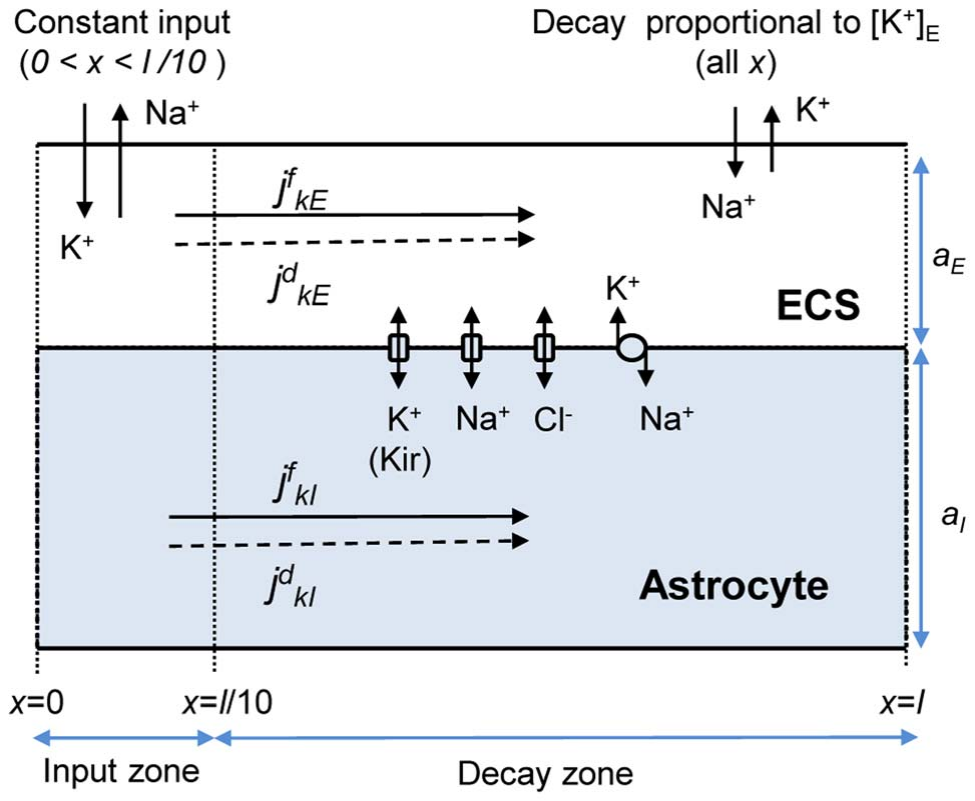
Astrocyte model. A representative astrocyte (*I*) exchanging ions with the ECS (*E*). As indicated, ions could cross the astrocytic membrane via passive Na^+^ or Cl^−^ channels, via the K^+^ Kir channel or the Na^+^/K^+^-pump. Ions could also be transported longitudinally by electrical migration 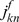 or diffusion 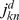 through the ICS (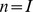) or ECS (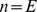). The cation-exchange input was a constant influx of K^+^ and efflux of Na^+^ to/from the ECS of the *input zone* (defined as the region 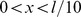). The cation-exchange output was an efflux of K^+^ and influx Na^+^ from/to the ECS. The output was proportional to the local K^+^-concentration, and occurred over the whole axis. The *decay zone* was defined as the part of the axis where no input was applied (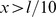), i.e., the region where there was a net efflux of K+ from the system.

We here present a model of astrocytes exchanging ions with the ECS, as sketched in Fig. 3, and defined in further detail below. The astrocyte model was developed for macroscopic transport processes, involving a collection of astrocytes (possibly connected via gap junction into a syncytium) in a piece of tissue. For this problem, we used the geometrical simplification motivated in Fig. 1, i.e., we applied the geometry in Fig. 1*B*. We took the intracellular domain 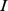 to represents a phenomenological “average” astrocyte (the cable, 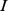), surrounded by a sheet of ECS (the coating, 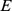). We used the empirical estimates that a fraction 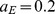 of neural tissue volume is ECS, while astrocytes take up a fraction of about 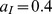 of the total tissue volume [12]. The intracellular domain was therefore twice as voluminous as the intracellular.

Table 1 contains a list of definitions that are necessary for the reader to follow the remainder of the paper. The dynamics in the system was due to fluxes of ions crossing the membrane 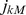, or axial fluxes in the ECS or ICS due to diffusion (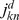) or migration in the electrical field (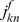). We assumed that only the three main charge carriers (K^+^, Na^+^ and Cl^−^) contributed to electrodiffusive transport. For the diffusion constants (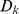), we used values valid for electrodiffusion in diluted media [36], modified with the tortuosities (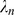) estimated in [12]. The same values have also been used in earlier, related studies [31], [37]. All relevant model parameters are listed in Table 2. The system input, and the astrocytic membrane mechanisms are defined in further details below.

**Table 2:** Model parameters.

Parameter	Value	Reference
*l* (length of astrocyte)	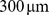	
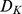 (K^+^ diffusion constant)	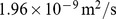	[31], [36], [37]
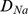 (Na^+^ diffusion constant)	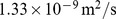	[31], [36], [37]
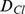 (Cl^−^ diffusion constant)	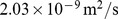	[31], [36], [37]
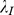 (intracellular tortuosity)	3.2	[12]
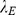 (extracellular tortuosity)	1.6	[12]
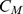 (specific membrane capacitance)	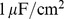	[38]
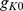 (baseline K^+^-conductance)	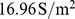	[38]
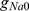 (baseline Na^+^-conductance)	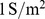	[38]
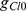 (baseline Cl–conductance)	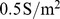	[38]
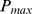 (maximum Na^+^/K^+^ pump-rate)	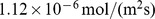	[20]
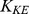 ( 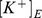 -threshold for Na^+^/K^+^ pump)	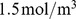	[20]
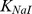 ( 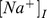 -threshold for Na^+^/K^+^ pump)	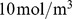	[20]
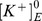 (initial ECS K^+^-concentration)	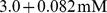 *	[20]
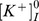 (initial ICS K^+^-concentration)	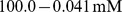 *	[20]
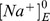 (initial ECS Na^+^-concentration)	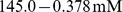 *	[20]
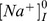 (initial ICS Na^+^-concentration)	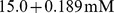 *	[20]
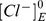 (initial ECS Cl–concentration)	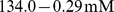 *	[20]
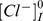 (initial ICS Cl–concentration)	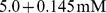 *	[20]
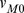 * (initial membrane potential)	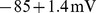	[20]
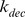 † (decay factor for 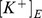 )	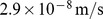	[54]
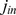 (constant input in input zone)	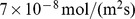	

* Initial concentrations are given as 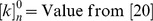
+ *Correction*, where the sum gives the baseline (resting) concentration in the default parametrization of the model.

†The maximum average Na^+^/K^+^-pump rate for a single neuron was estimated to 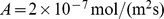 [54]. We obtained 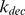 by solving 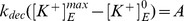, assuming that 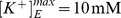.

#### Input/output

Our model system explicitly includes astrocytes and the ECS. Neurons were not explicitly modelled. However, we assumed that any external input to or output from this system reflects the activity of local neurons.

We were interested in simulating how astrocytes are involved in transferring K^+^ out from high concentration regions. To induce such a high-concentration region, a selected region (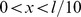) of the ECS, was exposed to a constant influx of K^+^ and (in order not to introduce any net charge to the system) a corresponding efflux of Na^+^:
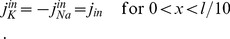
(30)The input mimics the effect of enhanced activity of local neurons, taking up Na^+^ and expelling K^+^ into the ECS, thus causing the local extracellular K^+^-concentration (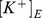) to rise. We refer to the region receiving the input as the *input zone*.

During normal conditions, neurons maintain their resting condition partly by uptake of K^+^ and release of Na^+^ via Na^+^/K^+^-exchangers. As opposed to the system input, this process would produce an efflux of K^+^ from the ECS of the model-system, and an influx of Na^+^. With reference to the K^+^-efflux, we refer to this process as the system output. Our model of the output differed from that of the input in two important ways: (i) Unlike the input, the output was applied over the full system axis (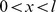), i.e., was contributed to by the highly active neurons in the input zone as well as normally functioning neurons outside this zone. (ii) Unlike the constant input, the output was assumed to depend on the local K^+^-concentration, causing 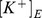 to decay towards the baseline concentration 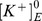:
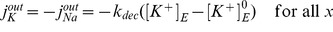
(31)The decay factor (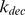) was set to a realistic value for maximal neuronal Na^+^/K^+^-exchange under physiological conditions (see Table 2). The input flux density reflected the activity level of local highly-active neurons. In our simulations, we specified 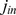 to a value that gave a K^+^-concentration of about 10 mM in the input zone during constant input (see Results for details). This concentration level is on the critical threshold between functional and pathological conditions [3], [12], [21], and should thus represent a case where the spatial buffering process plays a critical role.

We note that the distinction between an input and an output flux density had a practical motivation, as we wanted to to distinguish between processes causing K^+^ to enter/leave the system (we could instead have defined a net input as 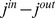). We also note that both the input and output were cation-exchanges, and thus did not introduce any net charge to the system (cf. Eq. 28).

#### Astrocytic membrane mechanisms

Four selected astrocytic membrane mechanisms were adopted from a previous point-model of an astrocyte [38]. The included mechanisms were standard, passive Na^+^ and Cl^−^ channels, the inward rectifying K^+^-channel (Kir), and the Na^+^/K^+^-pump, as sketched in Fig. 3. The transmembrane ion fluxes in the astrocyte model were:
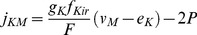
(32)

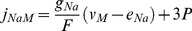
(33)

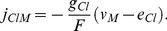
(34)Here, 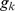 are the passive conductances of the K^+^ (Kir), Na^+^ and Cl^−^ channels. The currents depend linearly on the difference between 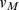 and the reversal potential,
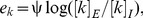
(35)for the respective ion types (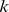). The potassium current was modified by the Kir-function [12]:
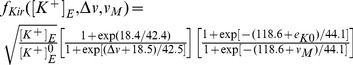
(36)where 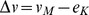, and 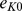 is the Nernst potential for K^+^ at basal concentrations 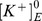 and 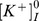.

The K^+^/Na^+^-pump uses energy (ATPase) to exchange 2 potassium ions with 3 sodium ions. We used a pump-rate per unit area defined by:
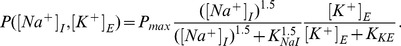
(37)The maximum pump rate, 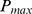, and the threshold concentrations, 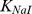 and 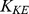, are given in Table 2.

#### Initial conditions

Initial conditions were determined in the following way: As a starting point, we used 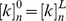 and 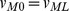 as our initial conditions, where 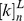 and 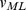 were the resting concentrations and resting membrane potential found in a previous study [20]. We then ran a simulation with no system input or output. With the membrane mechanisms included in Eqs. 32–34, the system had a simulated resting state (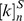 and 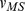) which was close to, but not identical with 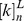 and 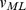. For all subsequent simulations, we set the initial conditions to the simulated resting conditions (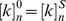 and 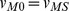). The estimated values and the values from the literature are given in Table 2.

Prior to all simulations, we defined the static charge densities:
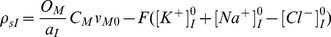
(38)

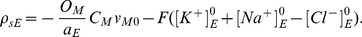
(39)The static charge densities ensure that the total charge density in 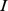 and 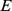 are consistent with 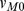, according to Eq. 7.

#### Comparison of concentrations and charges

To allow direct comparison with ion concentrations, we represent the charge density in Eq. 7 as an equivalent concentration of unit charges, defined by:
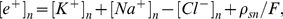
(40)with Eq. 38 or Eq. 39 for 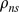. Likewise, we represent the current densities as equivalent unit-charge flux densities, defined by:
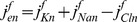
(41)

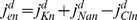
(42)


#### Implementation

**Figure 4:**
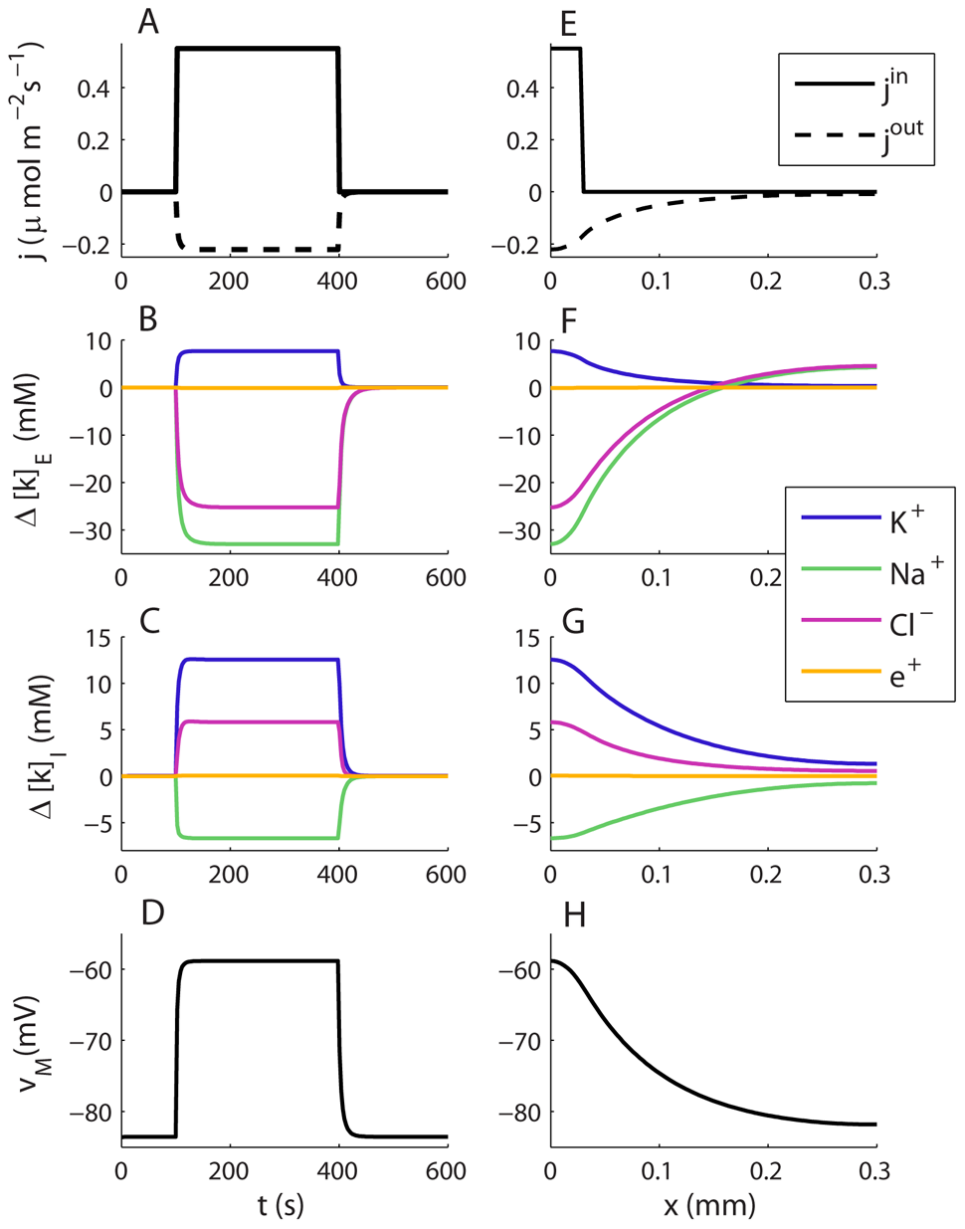
Dynamics and steady state profiles for the astrocyte/ECS-system. (A–D) Dynamics of selected variables in a point (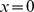) in the input zone. (E–H) Spatial profiles of selected variables at a time 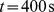, when the system was in steady state. The constant cation-exchange input was applied to the ECS of the input (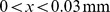) zone from 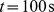 to 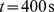. (A) The input and output flux densities of K^+^ to the point 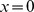. We recall that the Na^+^ input/output (not shown) was the opposite of that of K^+^: 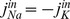 and 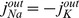. (B,D) During the input, ion concentrations in the ECS and ICS changed, but reached steady state after about 10–50 s after stimulus onset. (B) 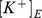 (at 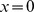) had then increased by about 7.7 mM with respect to the baseline value. (C) 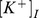 had increased by about 12.5 mM due to uptake by the astrocyte. (D) The astrocytic membrane potential had been depolarized to about −59 mV at 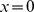. The impact of the input was smaller outside the input zone. (F–H) Deviations from the baseline ionic concentrations and 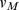 typically decreased with 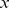. Far away from the input zone (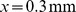), the conditions were close to the baseline conditions. (B–C, F–G) Ionic concentrations were represented in terms of deviations from resting concentrations: 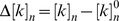 for 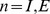. For direct comparison with ion concentrations, the charge density was represented as an equivalent concentration of unit charges 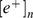.

**Figure 5:**
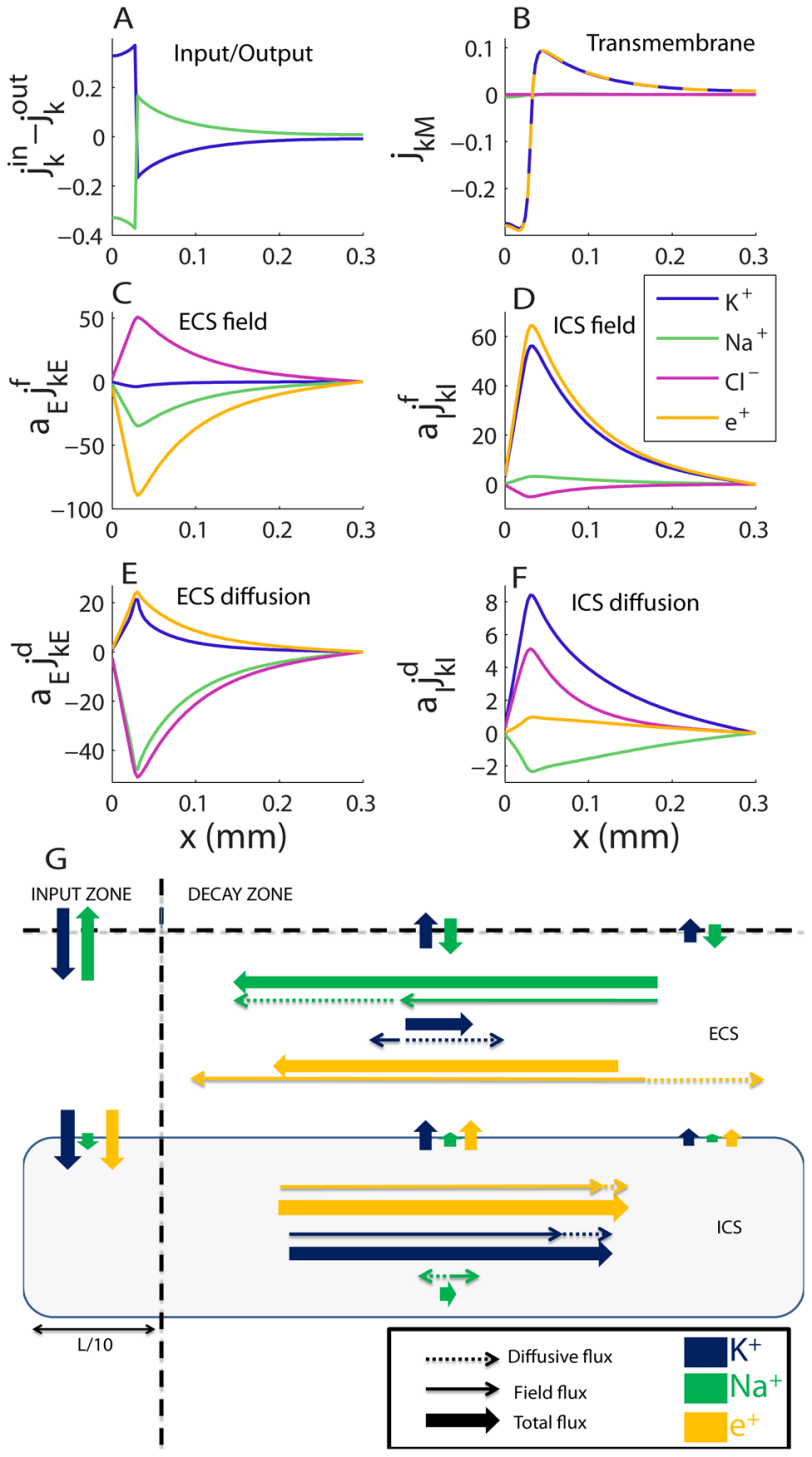
Transports in the astrocyte/ECS system during steady state. (A) Total flux densities into system (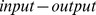). (B) Transmembrane flux densities. (C–F) Longitudinal flux densities due to (C) electrical migration in the ECS, (D) electrical migration in the ICS, (E) diffusion in the ECS and (F) diffusion in the ICS. (A–D) To aid comparison, flux densities 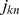 were scaled by the relative area fraction 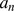 (e.g., if 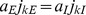, 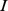 and 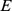 carry the same the net flux of ion species 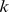). (G) A flow chart that qualitatively summarizes the essential information in (A–F), showing the main transport routes of K^+^ and Na^+^ during SS (Cl^−^ excluded from the overview). K^+^ generally entered the system in the input zone and left the system from some point along the astrocyte axis. The transport route of K+ (from entering to leaving the system) was predominantly intracellular, demonstrating the astrocyte's efficiency as a spatial buffer. Na^+^ entered in the decay zone and left from the input zone. Na^+^ transport predominantly took place in the ECS. The illustration (G) is qualitative - longer arrows mean higher flux densities, but the mapping from (A–F) to (G) is not quantitatively exact. The input zone was in the region 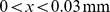. Units on the 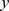-axis are 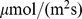 in all panels.

The model was implemented in Matlab, and the code will be made publicly available at ModelDB (http://senselab.med.yale.edu/modeldb). Simulations were run using the Matlab-solver *pdepe*, which uses variable time steps. For the simulations presented below, we used a maximum time step of 0.1 s, and used 100 segments in the 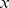-direction. A single simulation (e.g., producing Figs. 4 and 5) then took about 1 min to run on a standard laptop. Improving the resolution had no visible impact on the predicted results. Initial conditions were as listed in Table 2, and the sealed-end boundary conditions (Eq. 6) were applied.

## Results

An important contribution of this work was the general electrodiffusive formalism presented in the Model section. This formalism represents a framework for modeling the dynamics of the membrane potential (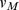), the intra- (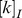) and extracellular (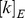) ion concentrations. The formalism is general to the choice of membrane mechanisms, and could be applied to model any transport process that justifies the geometrical simplification depicted in Fig. 1.

Here, we have applied the formalism to simulate spatial K^+^-buffering by astrocytes, using the specific implication to the atrocyte/ECS-model, also presented in the Model section. Our main objective has been to investigate the transport routes of K^+^ ions, from entering the system in the ECS of the input zone, to leaving the system at some point along the 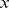-axis. We remind the reader that a useful list of symbols and definitions can be found in Table 1.

### Ion concentration dynamics in the Astrocyte/ECS system

We investigated the ion concentration dynamics in the astrocyte model (Fig. 3) in full detail. Fig. 4*A–D* shows the dynamics of selected variables in the input zone (at 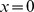). Fig. 4*E–H* shows how the same variables depend on 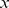 at a time 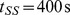 when the system was in SS. We explain this further below.

The input was applied from 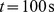 to 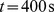 in the input zone (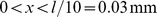). This is illustrated in Fig. 4*A* (solid line), which shows the flux density of K^+^ (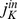) entering the system in the input zone. We recall that the input was a cation exchange, so that there was an equal flux density of Na^+^ leaving the system (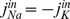). For simplicity, 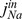 was not included in the figure, but we keep in mind that whenever K^+^ entered/left the system, an equal amount of Na^+^ left/entered. The cation-exchange input thus caused an increase in 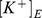 and a decrease in 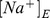 in the input zone. This can be seen in Fig. 4*B*. The notation 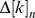 represents the deviations from baseline concentration (cf. Table 2).

As 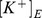 increased, the output from the system (being proportional to 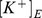) increased. Also this is illustrated in Fig. 4*A* (dashed line), which shows the flux density of K^+^ (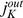) leaving the system from a point 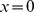 in the input zone. We recall that also the output was a cation exchange, so that the efflux of K^+^ implied a corresponding influx Na^+^.

The input was given in the input zone, while the output occurred over the full axis, depending on the local value of 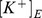. During a transient period, the constant input changed the ion concentrations in the system. The system reached steady state (SS) when 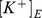 became sufficiently high. Then, the total amount of K^+^ entering the system per second, and the total amount of K^+^ leaving the system per second, coincided (with the same being true for Na^+^). This is illustrated in Fig. 4*E*, which shows how the 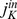 and 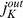 are distributed over the 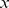-axis at a time 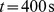, when the system was in SS. The areas under the curves for 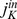 and 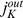 were then equal. In the input zone, however, the output rate was about 1/3 of the input rate (Fig. 4*A*). This means that about 2/3 of the K^+^ that entered the system was transported in the positive 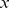-direction, and left the system from the decay zone. (We recall from Fig. 3 that the decay zone is defined as any part of the 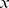-axis outside the input-zone).

Fig. 4*B–D* shows how the local (at 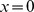) intracellular ion concentrations, the extracellular ion concentrations and 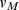 changed from the input had been turned on until the system reached SS. For the present example it took 49 s from the constant input had been turned on until the slowest variable (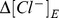) reached 99% of its SS value. The other variables approached SS faster than this (e.g., 12 s for 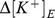 and 19 s for 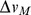). During SS, 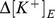 was about 7.7 mM, corresponding to a concentration 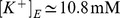 (as the baseline concentration was 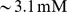). Although the input was applied to the ECS of the input zone, the local intracellular K^+^-concentration had increased even more (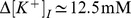). This reflects the astrocyte's propensity for local K^+^-uptake. The changes in ionic concentrations in the ECS and ICS coincided with a local depolarization of the astrocytic membrane, from the resting potential (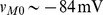) to about 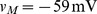, reflecting concentration dependent changes in the reversal potentials of the involved ionic species.

From here on, we focus on the SS-situation, i.e., on the activity of astrocytes during periods of on-going intense neural activity. For all system variables, the devition from the baseline (resting) conditions were generally biggest at the point 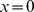, i.e., in the part of the input zone which is furthest away from the decay zone (Fig. 4*E–H*). The average value of 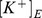, taken over the input zone (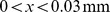) was approximately 10 mM (about 6.9 mM above the resting concentration). During the model calibration, the constant input rate (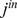) was tuned to obtain this value, which is on the threshold between functional and pathological conditions [3], [12], [21]. During SS, the gradients in ionic concentrations (Fig. 4*F–G*) and 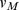 (Fig. 4*H*) were quite pronounced. We thus expect that both diffusive and electrical forces contribute to transporting ions through the system (from entering to leaving). This is explored further in the following section.

#### Ion transport pattern in steady state

Fig. 5 shows spatial profiles of all ionic flux densities during SS. As Fig. 5*A* shows, there is a net external influx of K^+^ (blue line) to the in the ECS of the input zone (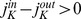), and a net external efflux of K^+^ in the ECS of the decay zone (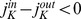). In the case of Na^+^, the situation is opposite.

We first focus on the transports of K^+^, from entering the system in the input zone (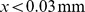), to leaving from some point along the 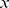-axis. From Fig. 5*B* we see that K^+^ is taken up by the astrocyte in the input zone (negative 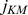 represents an inward flux density), and released from the astrocyte to the ECS in the decay zone. This implies that there must be longitudinal transport of K^+^ inside the astrocyte, out from the input zone. The longitudinal flux densities are shown in Figs. 5*C–F*. We have distinguished between field flux densities (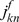), driven by voltage gradients, and diffusive flux densities (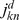), driven by concentration gradients (cf. Eq. 4). In the ECS, the electrical migration of K^+^ (Fig. 5*C*) was in the negative 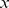 direction, while diffusion was in the positive 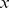-direction (Fig. 5*E*). Inside the astrocyte, diffusion and electrical migration were both in the positive 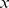-direction (Figs. 5*D, F*). Transport of K^+^ in the positive 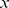-direction (out from the input zone) therefore had the best conditions in the ICS.

In the case of Na^+^, the situation was different. Firstly, Na^+^ entered the system in the decay zone of the ECS, and left the system from the input zone (Fig. 5*A*). The transmembrane Na^+^-flux was very small (Fig. 5*B*), and the main longitudinal transport occurred in the ECS. As in the case of K^+^, electrical migration of Na^+^ in the ECS, was in the negative 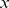-direction. However, for Na^+^, this was also true for diffusion. Longitudinal transport of Na^+^ therefore had good conditions in the ECS, as diffusion and electrical migration both drove Na^+^ in the same direction (towards the input zone).

The main transport routes K^+^ and Na^+^ during SS are summarized in Fig. 5*G*: K^+^ entered the system in the ECS of the input zone, where a major fraction of it crossed the membrane. Transport of K^+^ out from the input zone predominantly took place inside the astrocyte. Outside the input zone (i.e., in the decay zone), the astrocyte released K^+^ to the ECS, from where it eventually left the system. Na^+^, on the other hand, entered the system in the decay zone, and was predominantly transported longitudinally through the ECS before leaving the system from the input zone. The net Cl^−^ transport (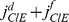) was very small (flux densities due to diffusion and electrical migration canceled each others out), and was not included in the summary.

Two basic mechanisms explain the qualitative difference between Na^+^ and K^+^ transports. Both are related to the membrane being most depolarized in the input zone (Fig. 4*F*). The first mechanism concerns the axial fluxes. As the astrocyte was most depolarized in the input zone, the charge density (positive in the ICS and negative in the ECS) had the highest absolute value there. Therefore, the electrical forces on K^+^ and Na^+^ (being cations) were in the negative 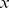-direction in the ECS (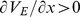), and in the positive 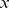-direction in the ICS (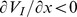). This favoured the ICS for transporting K^+^ away from the input zone, while it favoured the ECS for transporting Na^+^ into the input zone. Furthermore, this finding predicts that the astrocyte not only provides an additional and more effective domain for longitudinal K^+^-transport, but even reduces the net transport of K^+^ through the ECS. To our knowledge, we are the first to suggest that astrocytes may use this mechanism for shielding the ECS from K^+^.

**Figure 6:**
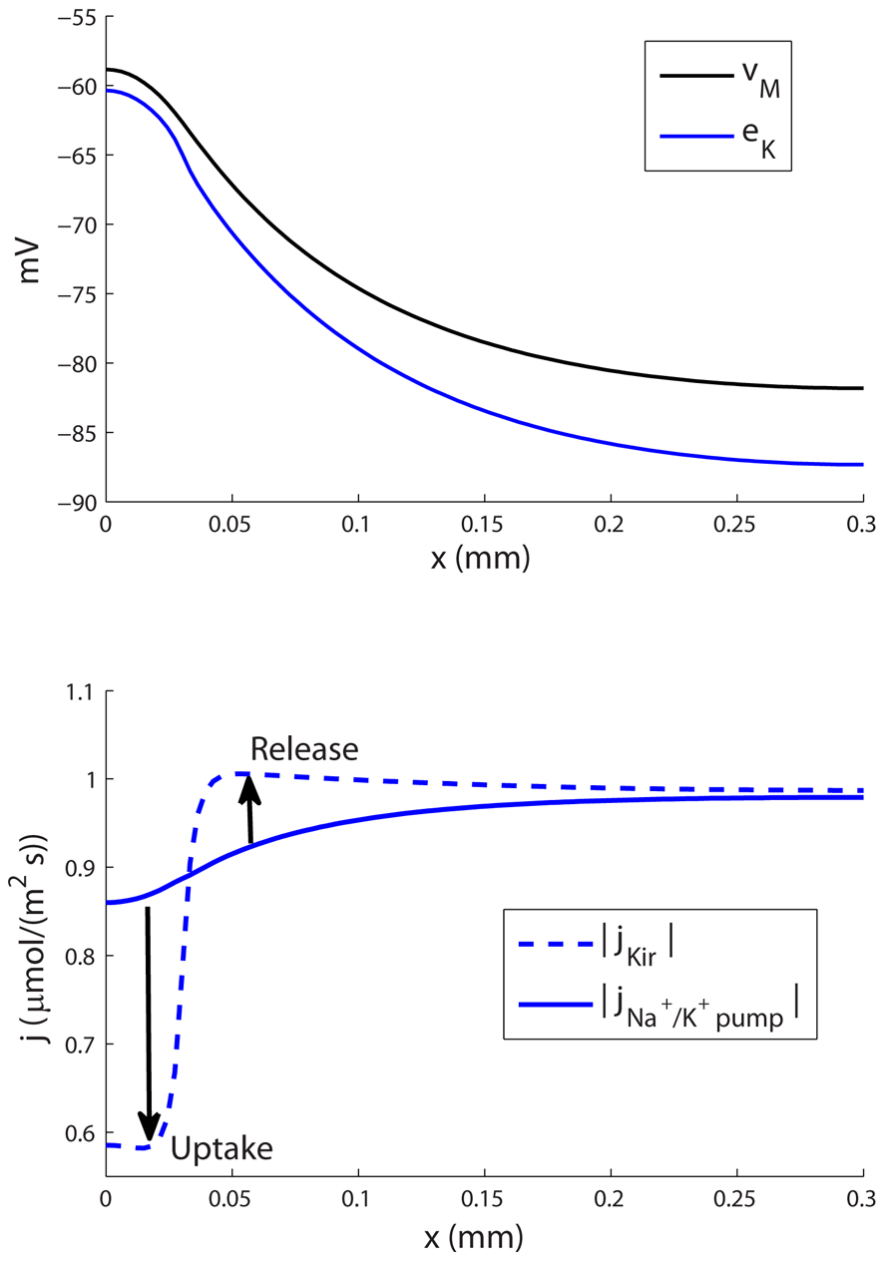
Membrane mechanisms involved in spatial K^+^-buffering. (A) The K^+^ reversal potential (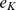) was more negative than 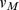 at all points along the 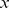-axis. The Kir-channel thus exclusively mediated an outward K^+^-current. (B) In the input zone 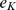 was close to 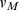, and the outward Kir-current was small compared to the inward current through the Na^+^/K^+^-pump. In the decay zone, the outward Kir-current was bigger, and dominated over the inward current through the Na^+^/K^+^-pump. Therefore, the astrocyte took up up K^+^ in the input zone, and released K^+^ in the decay zone (as indicated by arrows in (B)).

The second mechanism for explaining the differences between the Na^+^ and K^+^ transports concerns the transmembrane fluxes. The Na^+^/K^+^-pump mediated an inward flux of 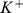 and an outward flux of Na^+^. Passive fluxes in the opposite direction (Na^+^ in through the passive Na^+^-channel, and K^+^ out through the Kir-channel), prevented further accumulation of ions inside the astrocyte. These passive fluxes were proportional to the deviation between 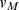 and the reversal potential (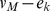). In the case of Na^+^, the passive flux and the pump rate were locally closely balanced across the length of the astrocyte (results not shown). The transmembrane Na^+^-flux was therefore small everywhere (Fig. 5*F*). During SS, the Kir-reversal potential was more negative than 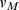 at all points along the 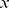-axis (Fig. 6*A*). Therefore, Kir exclusively conducted an outward K^+^-flux. However, this outward flux was small in the input zone, where the Kir-reversal potential 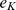 was close to 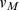. In the input zone, therefore, the Na^+^/K^+^-pump dominated, giving rise to a net K^+^-uptake by the astrocyte (Fig. 6*B*). Outside the input zone, 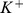-release through the outward Kir-channel dominated. These simulations support the prevailing view that in the context of spatial buffering, Kir mainly mediates an outward current, and that the main uptake is due to the Na^+^/K^+^-pump (see reviews in [3], [25]).

#### Sensitivity analysis

**Figure 7:**
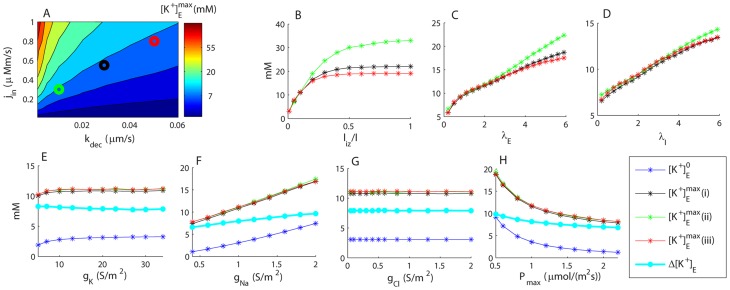
Sensitivity analysis. Sensitivity of 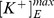 (maximal extracellular 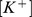 in the input zone) to variation in selected model parameters. (A) Sensitivity to input flux density (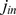) and the output rate constant (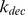). Similar values of 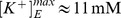 were obtained for the three marked data points: (i) black: 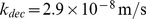, 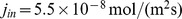 (default conditions), (ii) green: 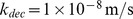, 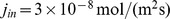, and (iii) red: 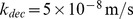, 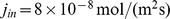. B–D) Sensitivity to the length of the input zone (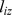), and tortuosities in the ECS (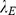) and ICS (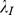). (E–H) Sensitivity of 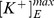 and 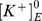 (baseline extracellular 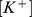) to membrane conductances (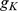, 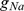 and 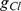), and the maximal Na^+^/K^+^-pump rate (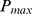). 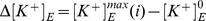. (B–H) The legend applies to all panels. Black (i), red (ii) and green (iii) lines correspond to the input-parameter combinations marked in (A).

The qualitative model performance was robust to parameter variations. Fig. 7 shows the sensitivity of the peak K^+^-concentration in the ECS during SS (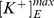) to variations in selected model parameters. All peak values occurred at 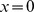.

As expected, 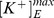 depended on the balance between the input rate (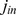) and decay factor (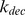). Fig. 7*A* shows that 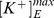 increased with increasing 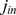 and decreased with increasing 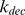. The default parameter values (see Table 2) are indicated with a black circle, while green and red circles indicate two other combinations of 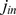 and 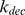 which gave similar peak amplitudes (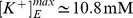) at 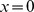.


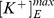
 increased smoothly with increasing input zone length (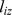) (Fig. 7*B*). The sensitivity to the tortuosities (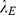 and 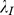) was also as expected (Fig. 7*C–D*). Increasing 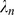 corresponds to decreasing the effective diffusion constant (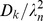), and thus had a negative impact on the system's ability to buffer K^+^ spatially.

Variations in the membrane parameters (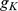, 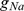, 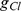 and 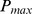) led to changes, not only in 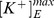, but also in the baseline concentration (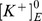) prior to the input (Fig. 7*E–H*). As long as the parameters were kept reasonably close (within 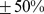) to the default parameter values, the system behaviour was qualitatively similar to that observed in Figs. 4 and 5. The sensitivity to 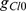 was low.

The high sensitivity to 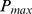 (Fig. 7*H*) can be understood quite intuitively: An increased Na^+^/K^+^-pump rate led to more K^+^ leaving the astrocyte, and thus an increased ECS concentration. The sensitivity to 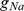 (Fig. 7*F*) has a more indirect interpretation: When 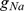 was decreased, the passive Na^+^ current into the astrocyte decreased, and the outward current through the Na^+^/K^+^-pump led to a hyperpolarization of the astrocyte. For example, with 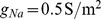, the resting membrane potential was 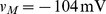. An equilibrium between K^+^ influx through the pump and efflux through the Kir-channel then required a corresponding hyperpolarization of the K^+^ reversal potential (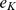), i.e., an increase of 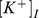 on behalf of 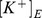 (cf. Eq. 35).

The sensitivity of 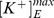 and 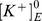 to variations in 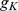 was low (Fig. 7*E*). This, somehow counterintuitive, finding was likely due to the Kir-channel being the most abundant membrane mechanism, with 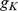 being about 17 times as high as 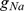 by default. Despite moderate variations of 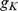, 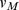 therefore always resided relatively close to 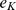 (results not shown). A low sensitivity to pharmacological intervention with astrocytic Kir-channels has also been found experimentally [39]. In our simulations, however, a further reduction of 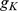 (below the parameter range in 7*E*), caused 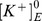 to drop rapidly towards 0, where the mathematical system is singular. We did not explore this effect further, but note that egn. 36 for the Kir-channel was empirically determined for retinal Müuller cells [12], [21], and it is questionable whether it is applicable at extreme parameter values (i.e., extremely low concentrations).

#### Electroneutrality

It has been previously withheld that to preserve electroneutrality, an influx of K^+^ from the ECS to the glia cell must be accompanied with an influx of an anion (such as Cl^−^) or an efflux of another cation (such as Na^+^) [30]. Physically, however, the system is not strictly locally electroneutral in the thin Debye-layer surrounding the capacitive membrane. Before the system reaches steady state, there must be a net transfer of charge into the astrocyte, consistent with the depolarization of the membrane.

A consistent relationship between 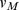 and ionic concentrations is implicit in the electrodiffusive formalism presented here. To get an insight in the relationship between 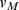 and ionic concentration, we have presented the charge density in Fig. 4 as an equivalent concentration of positive unit charges 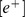 (cf. Eq. 40). In the astrocyte model, the resting potential 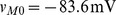 corresponded to concentrations 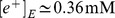 and 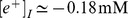 (the negative concentration of 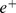 unit charges could be read as a positive concentration of negative unit charges 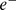.) Physically (although this is not explicit in a one dimensional model) this represents the charges stuck on both sides of the membrane. These are equal in magnitude, but have opposite signs (the concentrations differ by a factor 2 due to the ICS having twice the volume of the ECS). At SS, 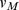 had increased from the resting potential to about −60 mV, consistent with small absolute changes (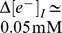 and 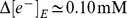) in the concentration of unit charges. As seen in Fig. 4*B–C*, these changes were very small compared to the changes 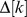 in any of the ionic concentrations. Anions and cations were thus always closely balanced in numbers, reflecting the nearly electroneutral nature of the system.

Only when the system had reached SS (and the capacitive current 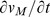 was zero), the net charge crossing the membrane was zero. However, this applied to the astrocyte as a whole, and was not locally true. During SS, there was a net influx of charge in the input zone due to the large uptake of K^+^ there. This is evident in Fig. 5*F*, where net electrical currents have been represented as equivalent flux densities of unit charges (cf. Eqs. 41–42). This did not lead to any accumulation of charge inside the astrocyte, as the charge that entering was transported intracellularly out from the input zone (Fig. 5*B,D*) before being re-released to the ECS in the decay-zone (Fig. 5*F*). During SS, the sum of the Na^+^ and K^+^ transports gave rise to a net electrical current which cycled in the system (Fig. 5*G*).

We also add a remark regarding the limitations of standard cable theory. In standard cable theory, diffusive currents are assumed to have a negligible impact on 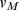, and intra- and extracellular resistivities are assumed to be constant (i.e., not dependent on ion concentration variations). During our simulations ionic concentrations changed by several mM before the system reached SS (Fig. 4). These changes corresponded to a 10% decrease and 20% increase, respectively, in the intra- and extracellular resistivities (cf. Eq. 15, results not shown). Furthermore, Fig. 5 showed that the diffusive current contributed quite significantly to the net electrical transport, and was about 25–30% of the field current in the ECS. Hence, for the process simulated here, standard cable theory would give relatively poor predictions of 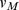.

### The relative importance of spatial buffering in K^+^-clearance

**Figure 8:**
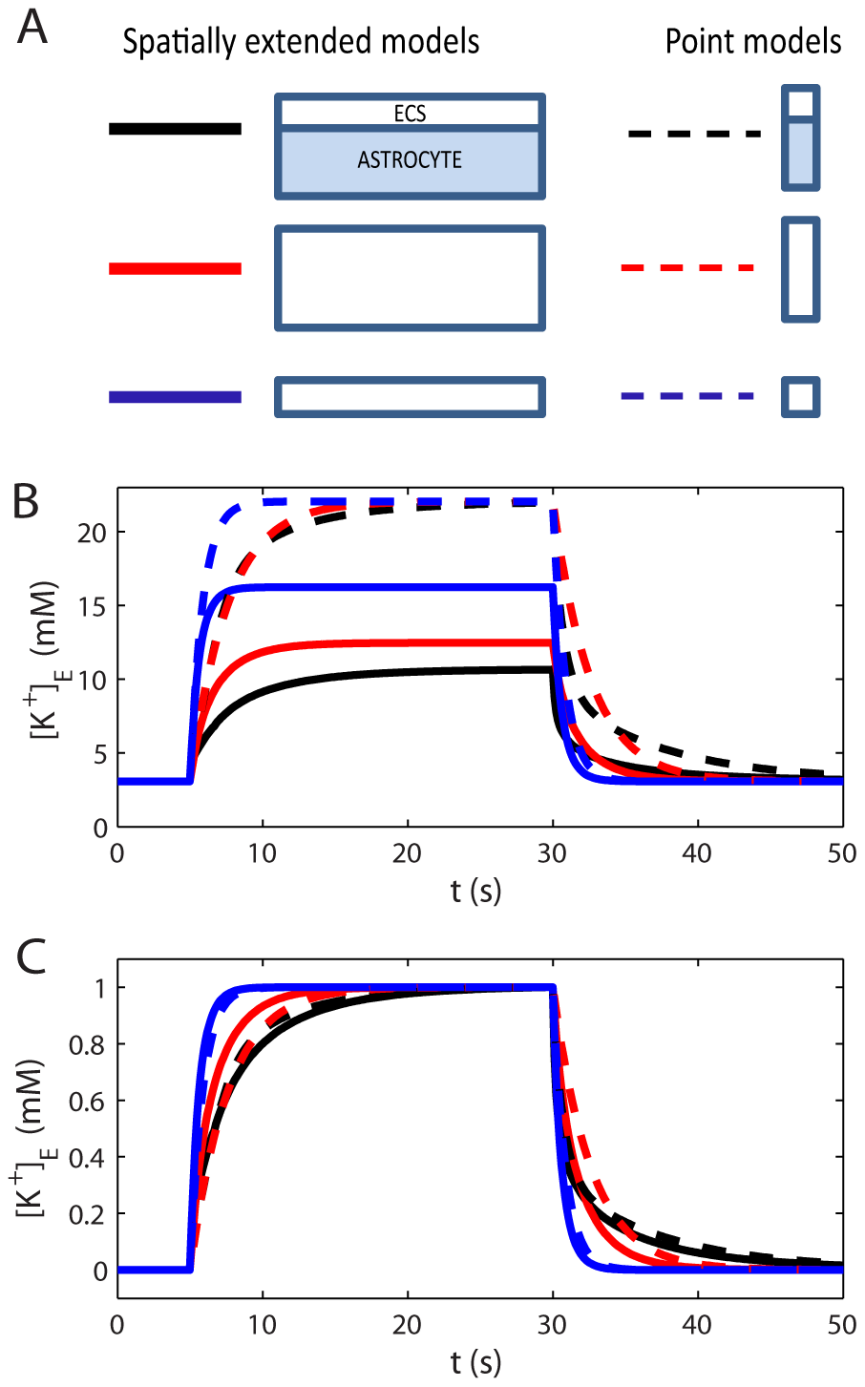
Model comparison. (A) Six model versions, three spatially extended models (solid lines), and three point models (dashed lines). Two versions (black lines) included an active astrocyte. In two versions (red lines), the astrocyte volume had been exchanged with an enhanced ECS (the total ECS volume fraction increased to 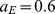). In two versions (blue lines), the original ECS volume fraction (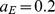) was kept when the astrocyte was removed. (B) The performance of the six model versions were compared in terms of maximal 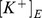 in the input zone during a constant K^+^ influx to the system. (C) To compare the time course of the 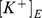 dynamics, the responses (in B) were normalized to the peak amplitude for each respective trace.

In addition to spatial buffering, K^+^ may also be buffered by diffusion through the ECS alone, or by local (space independent) storage by the glial cell, to be later released in the same region of the ECS [19], [24]. To investigate the relative importance these clearance mechanisms, we compared the 6 six model versions depicted in Fig. 8*A*, including one group of three spatially extended models (solid lines), and one group of three point models (dashed lines). Both groups included one model version with an active astrocyte, one model version where the astrocyte had been replaced by a corresponding increase in the ECS volume (the total ECS volume fraction increased to 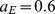), and one version where the original ECS volume fraction (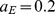) was kept when the astrocyte was removed. The spatially extended model including the astrocyte, is the one we studied in the previous sections. The other models were reduced versions of this.

All model versions were exposed to the input signal described by Eq. 30, causing an increase in 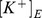. The input was applied in the time window 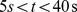, which was sufficient for 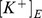 to reach its SS-value in all models. Fig. 8*B* shows the dynamics of the K^+^-concentration in the ECS at the point where the concentration was the highest (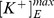). In the spatially extended models, this occurred at 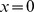, i.e. in the part of the input zone furthest away from the decay zone.

During SS, the net K^+^ efflux and influx from/to the system coincided. For the point models, having no spatial resolution, there was no distinction between the input zone and decay zone, as the input and output were injected to/subtracted from the same single compartment. The net output rate thus depended on 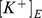 in this single compartment. Therefore, all point models approached the same SS value (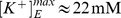). For the spatially extended models 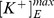 was lower, as parts of the K^+^ could leave the system also outside the input zone. For these models, 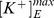 depended on how efficient they were in longitudinally transporting K^+^ out from input zone before (revisit Fig. 4 for more details).

To gain insight in the importance of local K^+^-uptake by astrocytes, relative to diffusion in the ECS, we compared the performance of the point model including the astrocyte (black, dashed line in Fig. 8*B*) to that of the spatially extended model including only the ECS (blue, full line). During the first few seconds after the stimulus had been turned on, the point model with the astrocyte (representing local uptake) was most efficient in terms of limiting 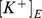. However, local uptake was limited by the storing capacitance of the astrocyte. After seconds with constant K^+^-influx to the system, the spatially extended model (representing diffusion through the ECS) performed better, as it could redistribute K^+^ over a larger spatial region. The astrocyte's ability to locally store excess K^+^ has been emphasized in previous investigations [19], [24]. Our simulations predicted that the local storage mechanism is mainly important in relatively short time spans after potassium release (a few seconds). A similar conclusion was also drawn from previous modelling studies [10], [12]. We here add an additional point to this discussion: The performance of the point model with extended ECS (dashed red lines) more or less coincided with that of the point model including the astrocyte (dashed black lines). In terms of local storage, the astrocyte (with its membrane being highly permeable to K^+^), essentially just acts to expand the local volume that the incoming flux of K^+^ enters into.

It has been argued that because K^+^-transport is aided by transmembrane processes as well as internal processes in the glial cell, K^+^ can be cleared more effectively by glia than would be possible by a much enlarged extracellular space [40]. To investigate this claim, we compared the three spatially extended models (solid lines). We found that the model including the astrocyte (black, solid line) was more successful in limiting 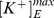 than any of the other model versions. It was significantly more successful than diffusion in the ECS alone, even in the (rather hypothetical) system where the extracellular volume had been increased by a factor 3.

In conclusion: In terms of local storage, the astrocyte was not significantly more efficient than an increased enlarged extracellular space. In terms of spatial buffering, however, it was.

#### Consistency of formalism

In all our simulations, 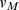 was defined in terms of the charge density in 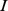, and computed algebraically by solving Eq. 19 at each time step. Identical results (down to a very small numerical error) were obtained when 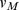 was defined by the charge density in 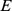 (Eq. 20), and when 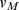 was computed differentially by using Eq. 29 (results not shown). As all transports are included in Eq. 29, the algebraic and differential methods yielded consistent results.

When the input was turned on and off, a small numerical error was introduced in the conservation of ionic concentrations, inducing a small error in the total charge in the system. The relative deviation from global charge neutrality (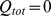), defined as 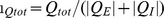, where 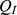 and 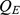 refer to the total charge in 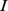 and 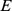, was about 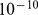. This gave rise to a relative deviation from perfect charge symmetry (cf. Eq. 18), defined as 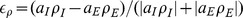, which was also on the order of 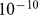 (for all 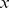). Accordingly, 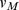 computed from the charge density in 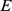 deviated by a relative factor 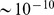 from 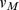 computed from the charge density in 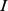. This corresponded to an absolute difference of 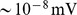. Errors were larger, but still small, when the differential method was used. Then 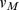 deviated locally by up to 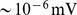 from 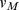 derived from the charge density in 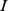 or 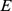.

Errors will generally depend strongly on the algorithm used for solving the differential equations, the time step, and the number of compartments in the simulated system. The errors could likely be reduced by using a smoother input signal than the step function in Eq. 30. We did not engage in further analysis of the origin of the errors, as we were content with their smallness in relation to the questions addressed here.

## Discussion

We presented a one-dimensional, electrodiffusive framework for modeling the dynamics of the membrane potential (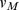) and the ion concentrations 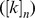 of all included ion species 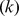 in an intra- and extracellular domain (Fig. 2). The framework could have a broad range of applications within the field of computational neuroscience. In the current work, it was applied to simulate the role of astrocytes in K^+^-removal from high concentration regions.

### Spatial K^+^-buffering by astrocytes

The astrocyte/ECS-model provided a mechanistic understanding of how astrocytes may remove K^+^ from high-concentration regions. In summary, the model astrocyte responded to a local extracellular increase in 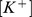 by a local depolarization of the membrane. At the same time, this depolarization (i) increased astrocytic K^+^ uptake in the input zone, (ii) increased astrocytic K^+^-release outside the input zone, (iii) decreased axial K^+^ transport in the ECS, and (iv) increased axial K^+^ transport inside the astrocyte. Furthermore, by comparing different versions of the model, we predicted that (v) local storage of K^+^ by astrocytes may play an important role at a short time scale, while (vi) at a longer time scale, the ability to distribute K^+^ spatially will be crucial for maintaining a low extracellular K^+^-concentration. In this regard, we found (viii) that the astrocyte was more efficient spatial buffering mechanism than diffusion in an enlarged extracellular space.

The findings (i–ii) were due to well documented astrocytic membrane mechanisms that we implemented in the model. Uptake from high concentration regions was mediated by the K^+^/Na^+^-pump, while release into low-concentration regions of the ECS was mediated by the Kir-channel. This supports the dominant view of glial K^+^-buffering [2], [3], [25], [39], [41].

The findings (iii–iv) were due to an interplay between electrical and diffusive forces. When locally depolarized (in the region with high extracellular 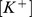), longitudinal voltage gradients arose, and ions in the ICS and ECS were exposed to an electrical force. In the ICS, the electrical force and diffusive force both drove K^+^-ions in the same direction (out from the high concentration regions). In the ECS, the electrical force acted in the opposite direction from the diffusive force, and reduced the net longitudinal transport through the ECS. Hence, in addition to being an efficient transport route for K^+^ out from high-concentration regions, the astrocyte actively reduces the extracellular K^+^-transport. This represents a (to our knowledge) novel mechanism that astrocytes may utilize to shield the extracellular space from excess K^+^. All these effects (i–iv) taken together turned the astrocyte into an efficient sluice for removing K^+^ from the input zone.

The findings (v–viii) shed light on the relative efficiency of spatial buffering and other K^+^-clearance mechanisms, such as local storage by astrocytes, or diffusion in the ECS alone. An interesting prediction was that, in terms of local storage, the astrocyte did not have a stronger effect on 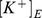 than an enlarged extracellular space would. In terms of longitudinal transports, however, the astrocyte performed much better (by spatial buffering) than diffusion in an enlarged extracellular space (Fig. 8). We do, however, wish to comment that these mechanisms are not mutually exclusive. In fact, an (initial) local accumulation of intracellular K^+^ is required for the astrocyte to initiate the spatial buffering process. It is this local accumulation that evokes the intracellular voltage- and concentration gradients that the astrocyte utilizes for intracellular K^+^-transport.

It is likely that the mechanisms responsible for spatial buffering vary between brain regions and between different species of glial cells [30]. Previous literature has suggested several mechanisms for spatial buffering apart from the ones that were included in our model. K^+^-uptake by Na^+^K/K^+^/Cl^−^-cotransporters and K^+^/Cl^−^-cotransporters are two candidate mechanisms that likely could affect the simulated results [38]. Furthermore, regions in the endfoot processes of astrocytes have been shown to have an extremely high K^+^-conductance compared to the membrane in general [42]. Such high concentration regions could improve the buffering process by transferring (siphoning) excess K^+^ into the vitreos humor or vasculature [43]. The buffering process may also be affected by water influx and swelling experienced by the astrocyte during the uptake process [13], [30], [38].

Rather than increasing the biological complexity, by e.g., including multiple candidate buffering mechanisms, we have in this study strived towards elucidating the fundamental physical processes involved in spatial K^+^-buffering. As our simulations demonstrated, K^+^-buffering is a highly complex process. It involves an intricate and sensitive interplay between 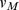 and ionic concentrations, and between electrical and diffusive transports. We therefore highlight the importance of applying an electrodiffusive, physically consistent, modelling scheme which ensures a complete book-keeping of ion concentration dynamics and its effects on 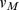. In previous models of spatial buffering, 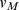 was derived from standard cable theory [10]–[12], [21], where diffusive currents are assumed to have a negligible impact on 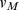, and where the resistivity is assumed to be constant (i.e., not dependent on ion concentration variations). During our simulations, intra- and extracellular resistivities changed by as much as 10% and 20%, respectively, and the diffusive current was about 25–30% of the field current in the ECS. The assumptions underlying standard cable theory are therefore poorly justified if applied to the spatial K^+^-buffering process.

### Macroscopic transports vs. single cell models

The astrocyte/ECS-model was represented phenomenologically as a single astrocyte coated with the average proportion of available ECS per astrocyte (see Figs. 1 and 3). This geometrical representation is justified for macroscopic transport processes, when a large number of astrocytes perform the same function simultaneously [12]. For the current study, this was a reasonable assumption, as the input was a change in the ion-concentrations in the ECS, shared by all present astrocytes.

If we instead wanted to study a cell specific signal, such as e.g., the response of a single astrocyte to a transmembrane current injection, the geometrical representation in Fig. 1*B* would be less appropriate. Firstly, the notion of the ECS as a relatively thin coating following a single cell is only motivated at the macroscopic “average transport”-level. Secondly, if only a single cell was involved in a particular process, we would expect that 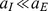. That is, a single active cell would have a significantly larger proportion of the ECS to its own disposal, compared to the macroscopic case, where all cells in a piece of tissue are active, and share the limited amount of available ECS. In single-cell models it is common to assume that conditions in *E* are constant, so that only *I* is modeled explicitly. In this limit, the electrodiffusive formalism reduces to the one-domain model presented previously by Qian and Sejnowski [31].

### Relationship to other electrodiffusive modeling schemes

The framework presented here is essentially an expansion of the one-domain model by Qian and Sejnowski [31] to a two-domain model that includes both the ECS and ICS. Like the one-domain model, the framework ensures (i) a consistent relationship between 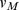 and ionic concentrations 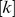. Unlike the one-domain model, the framework ensures (ii) global particle/charge conservation, and (iii) that the charges on either side of a piece of membrane must be equal in magnitude and opposite in sign (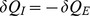). The latter constraint is implicit when the the membrane is assumed to be a parallel plate capacitor, an assumption made in most models of excitable cells (see e.g., [26], [27], [31]). It is also related to the topic of electroneutrality.

Electroneutrality in electrodiffusive models of biological tissue has been the topic of many discussions [44]–[46]. It is relevant for how the electrical potential (*v*), occurring in the Nernst-Planck equation, is derived. Generally (at sufficiently course spatial resolutions so that the charge density can be assumed to be continuous), *v* obeys Poisson's equation:
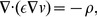
(43)where 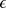 is the dielectric constant, and 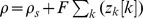 is the total charge density.

In biological tissue, the charge relaxation time 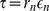 is very small in any region except in the thin Debye layer (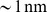) surrounding a bio-membrane. Any nonzero net charge density in the bulk solution will decay very rapidly (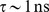) to zero [36]. Several models have simulated electrodiffusion by solving the Nernst-Planck equations in one or more dimensions, with Poisson's equation for 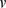 (see e.g., [45], [47]–[50]). The advantage with such a procedure is that the Poisson-Nernst-Planck (PNP) equations can be implemented in a general way in three-dimensional space. The challenge is then to specify the appropriate boundary conditions for solving Eq. 43 in the vicinity of membranes. Generally, PNP-solvers apply a fine spatial resolution near the membrane, and simulation time steps smaller than the charge-relaxation time [48]. For these reasons, they tend to be extremely computationally demanding [51].

The formalism presented in this work belongs to a class of of one-dimensional models, including the cable equation and several electrodiffusive models [10], [12], [13], [17], [31], [52], [53], which bypasses the computationally heavy PNP-scheme. The physical interpretation of these models is as follows: Any net charge in a volume 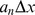 is implicitly assumed to be located in the thin Debye-layer surrounding the capacitive membrane, and is identical to the charge that determines 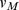. The remainder of the space (i.e., the bulk) will therefore be electroneural (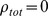). Note that any finite volume, enclosing a piece of membrane, will also be electroneutral. This follows from the charge symmetry condition (Eq. 18), constraining the charge on either side of the membrane to be equal in magnitude and opposite in sign. The charge symmetry condition and the electroneutrality condition are in this way closely related. In these electroneutral models, charge relaxation is implicit. This is a plausible assumption at time scales relevant for most biophysical processes. Accordingly, simulations may be run with time-steps ranging from 1 ms to 1 s, depending on the time course of the included membrane mechanisms.

### Final remarks

To our knowledge, the formalism summarized in Fig. 2 is the first biodiffusive model where the intra- and extracellular voltage gradients have been derived from the charge symmetry condition. Eqs. 25 and 26 can be interpreted as summarizing all local and global electrical forces driving the system towards electroneutrality. A natural future ambition would be to generalize the electrodiffusive formalism to 2 or 3 spatial dimensions, so it can address the same 3-dimensional transport problems as PNP-solvers. The challenge will be to formulate the system as a grid of coupled constraints (electroneutrality in the bulk and Eq. 12 for 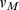 across the membrane) for which the Nernst-Planck equations can be solved with time steps much longer than those involved in the charge relaxation process.
